# Radiofrequency Electromagnetic Field Emissions and Neurodevelopmental Outcomes in Infants: A Prospective Cohort Study

**DOI:** 10.7759/cureus.87671

**Published:** 2025-07-10

**Authors:** Maninder S Setia, Revathi Natesan, Parineeta Samant, Sabrina Mhapankar, Sushil Kumar, Indra Vijay Singh, Apoorva Nair, Bageshree Seth

**Affiliations:** 1 Epidemiology, Mahatma Gandhi Mission (MGM) Institute of Health Sciences, Navi Mumbai, IND; 2 Pediatrics, Mahatma Gandhi Mission (MGM) Medical College and Hospital, Navi Mumbai, IND; 3 Biochemistry, Mahatma Gandhi Mission (MGM) Institute of Health Sciences, Navi Mumbai, IND; 4 Obstetrics and Gynaecology, Mahatma Gandhi Mission (MGM) Medical College and Hospital, Navi Mumbai, IND; 5 Pediatric Medicine, Mahatma Gandhi Mission (MGM) Medical College and Hospital, Navi Mumbai, IND

**Keywords:** ages and stages questionnaires, cell phone towers, cohort study, neuro-development outcomes, radiofrequency electromagnetic fields

## Abstract

Purpose

It has been argued that children are particularly at risk of developing health effects due to the emitted radiofrequency electromagnetic fields (RF-EMF). We designed this cohort to measure the association between exposure to RF-EMF radiation and neurodevelopmental changes in neonates and infants.

Methods

We present an analysis of 261 observations from a cohort of 105 neonates. The cohort was formed of pregnant women, and the neonates born to these women were followed for a period of one year. We assessed the level of radiation in the house using the Selective Radiation Meter 3006 (Narda Worldwide, Germany) and neurodevelopment using the Ages and Stages Questionnaire® (ASQ®)-3. We used random effects models for multiple observations in the same individual, and the main explanatory variable was household radiation levels (divided into tertiles as low/medium/high).

Results

The median (interquartile range (IQR)) range of radiation in all the households was 8.66 (IQR: 1.58, 23.11) mW/m^2^. It was 0.62 (IQR: 0.43, 1.58) mW/m^2^ in the lowest tertile, 8.66 (IQR: 5.00, 10.78) mW/m^2^ in the middle tertile, and 32.36 (IQR: 23.11, 45.60) mW/m^2^ in the highest tertile (p=0.0001). The mean scores were significantly lower in the middle and higher tertiles of LOR for the gross motor, fine motor, and problem-solving domains. The odds of children classified as ‘monitor/refer’ was significantly higher in the ‘high radiation group’ compared with ‘low radiation group’ for the fine motor (adjusted odds ratio (aOR): 2.74, 95% CI: 1.10, 6.78; p=0.03) and problem-solving domains (aOR: 3.67, 95% CI: 1.41, 9.55; p=0.008). We also found that low birth weight babies were significantly more likely to be classified as ‘monitor/refer’ for fine motor (aOR: 4.19, 95% CI: 1.73, 10.14; p=0.001), and problem solving (aOR: 2.57, 95% CI: 1.08, 6.13; p=0.033) domains.

Conclusions

Even after adjusting for low birth weight, we found that higher levels of radiation were associated with poorer outcomes for cognitive domains of development such as problem solving, and personal-social areas. Thus, there is a need to monitor the neuro-development of children in whom the RF-EMF radiations are expected to be higher (such as very close to cell phone towers, too many gadgets in the house).

## Introduction

An estimated 7.26 billion cell phone users have been reported all over the world at the end of 2022, and mobile phones form an important part of communication [1,2]. The popularity of cell phones has particularly increased among children and adolescents. A multi-country survey reported that 69% of children used mobile phones; the proportion was highest in Denmark (93%) and lowest in Japan (58%) [3]. A large proportion of children start using cell phones by the age of one year, and parents themselves give cell phones to the children while doing household work, at bedtime, and to keep them calm [4]. Another study found that the overall exposure to mobile phones was 76% in children less than five years of age; of these, about 21% were less than one year old and 25% were one- to two-year-olds [5].

Epidemiological studies have discussed the potential risk of these radio-frequency electromagnetic fields (RF-EMF) in various health outcomes such as increased risk of cancer and reproductive system and nervous system disorders [6,7]. Similarly, information is available on the harmful effects of radiation from cell phones and phone towers on the menstrual cycles, fertility, sleep disturbances, lack of concentration, and DNA damage (genotoxicity) [8-11]. It has been argued that children are particularly at risk of developing health effects due to the emitted radiation. Since children have a developing nervous system with higher water content and ion concentration, their brain tissue is more susceptible to impairment due to the energy emitted from the cell phones held next to the head than that of adults. About twice as much mobile phone energy is absorbed in the peripheral brain tissues of children as compared to adults [12]. Studies have also been conducted to assess the effect of RF on nervous system; these have reported an alteration of passive avoidance behavior and hippocampus morphology, decreased locomotor activity, tendency toward increased basal corticosterone levels, reduced memory functions, impaired cognitive performance, impairment of learning, and an associated change in acetylcholine receptor levels, decrease in cholinergic activity leading to short-term memory deficit, and changes in monoamine neurotransmitters [13-18].

Clinical and epidemiological studies and reviews have provided mixed results. Divan and co-workers reported that less than 5% of children had developmental delays at 6 and 18 months of age; they did not find a significant association between prenatal cell phone use and motor or cognitive/language developmental delays among infants at 6 and 18 months of age [19]. Furthermore, Kwon and colleagues also did not report any statistically significant effects on the neural change-detection profile [20], and Vrijheid and colleagues and other authors found little evidence of an adverse effect of maternal cell phone use during pregnancy and the neurodevelopment of offspring [21,22]. In another study, Divan and co-workers also reported that exposure to cell phones prenatally and, to a lesser degree, postnatally, was associated with behavioral difficulties such as emotional and hyperactivity problems around the age of school entry. Further, they concluded that these associations may be non-causal and may be due to unmeasured confounding [23]. However, few studies have assessed the association between levels of household radiation and outcomes in infants.

The Ages and Stages Questionnaire is a parent-reported screening questionnaire that assesses developmental milestones [24]. It is considered to be a useful screening tool to identify children at risk of developmental delay (five domains of the questionnaire) and detect severe disability in these infants, particularly in low- and middle-income countries [25]. In addition, the Ages and Stages Social Emotional Questionnaire is useful for screening the social-emotional competence of children [26]. We designed this cohort to compare these neurodevelopmental outcomes according to the severity of RF-EMF exposure in houses. We also wanted to study the factors associated with these outcomes in these children.

## Materials and methods

The present study is an analysis of data from 261 observations of a cohort of 105 neonates from Navi Mumbai, India.

Study population and procedures

The cohort was formed of neonates born to women who gave birth in our center. These women registered in our antenatal clinic in the first trimester of pregnancy and were followed according to the protocol of our center. All consecutive consenting women who were >18 years of age were eligible for inclusion. We excluded women who conceived after in-vitro fertilization, women detected as HIV-infected, or women positive for any other infection (such as hepatitis B virus (HBV), hepatitis B virus (HCV), syphilis, or the TORCH (toxoplasmosis, other (including syphilis, varicella-zoster, parvovirus B19, and sometimes HIV), rubella, cytomegalovirus (CMV), and herpes simplex virus) group of infections); women who have a current history of tuberculosis and in on anti-tuberculosis treatment; and neonates born with a congenital anomaly at birth were excluded from further follow-up. In the present analysis, we have presented data only from the neonates and infants. We assessed the radiation parameters and the neurodevelopmental parameters in these babies.

Radiation Parameters

We measured the cell tower properties close to the place of residence. We assessed the following parameters: 1) height from ground; 2) number of antennae in each base station; 3) down-tilt of the base stations (mechanical tilt as well as electrical tilt); 4) nature of the tower - whether it is a high gain or a low gain tower; 5) whether the base station is focused in a particular sector or is it omni-directional; and 6) transmitter power and effective radiated power. We then measured the household level of radiation of each individual included in the cohort. The radiation was measured using a Selective Radiation Meter 3006 (Narda Worldwide, Germany) [27]. The radiation for this component of the analysis was measured once at baseline by visiting their houses. The team members set up a prior appointment to visit the house. Though the exact time varied at each house, most of the appointments were usually made between 11 am and 3 pm. The team consisted of a scientific officer (Engineer) who was trained to measure the radiation. The radiation was assessed keeping all the activities in the household as it is (for example, if the Wi-Fi router remains on, then it was on when the radiation was assessed).

Neurodevelopmental Parameters

We used the Ages and Stages Questionnaire^®^ (ASQ^®^)-3 to assess the neurodevelopmental parameters in the infants. As stated earlier, this screening tool is important to identify children at risk for developmental delays and those who require monitoring and further referral services [25,28,29]. We followed the infants from two months onward. They were encouraged to follow up at two-month intervals (some of the follow-up visits by the mothers were planned to coincide with the vaccination schedule of their babies or regular health follow-up), and we used the age-appropriate measurement ASQ® form. This was to get as many observations from each infant as possible. The ASQ® has a sensitivity of 86% and a specificity of 85% [30]. It has five different scales - gross motor, fine motor, communication, personal social, and problem solving. We also used the ASQ®-Social Emotional (ASQ®-SE) Questionnaire. As indicated earlier, this is also a useful screening tool to evaluate social and emotional competence [26]. It was administered by a trained professional in outpatient settings.

We also collected information about the socio-economic status (SES) and detailed birth history (Apgar score at birth, birth weight) and included them in the present analysis. The socio-economic status was assessed using the Kuppuswamy scale. This is specifically constructed for the Indian population and is based on the education, occupation, and household income. A summary score is generated based on these three parameters, and the SES is classified as upper, upper middle, lower middle, upper lower, and lower [31].

Power Analysis

Since this is an interim analysis from a cohort, we have provided the power analysis for outcomes in the study. In our study, the power to detect a significant difference between the score for fine motor among the highest radiation tertile and the lowest radiation tertile was >90% (alpha=0.05, delta=-4.30). Similarly, the power to detect the difference in scores for gross motor was >90% (alpha=0.05, delta=-4.50), and it was >90% for problem solving (alpha=0.05, delta=-4.40).

Statistical methods

Data were entered in Microsoft Excel (Microsoft Corporation, Redmond, USA) and Stata Version 17 (StataCorp, College Station, Texas, USA). We estimated the means and standard deviations (SD), or medians and interquartile ranges (IQR) for linear variables. The medians across multiple groups were compared using the Kruskal-Wallis test. The means across multiple categories were compared using analysis of variance with post-hoc comparisons. We estimated the proportions for categorical variables. The proportions across multiple groups were compared using the chi-square test or Fisher’s exact test for low expected cell counts. A new variable - category of LOR - was created based on tertiles of the actual value of the household LOR. The three categories in this variable were low, medium, and high.

For multivariate analysis, we used random effects models for multiple observations in the same individual. These models not only account for between-individual correlation but also within-individual correlation and are a useful alternative compared with the standard logistic regression models. The main outcome for these random effects logistic regression models was monitoring/referral vs normal for the ASQ-3 and ASQ-SE scales, and the main explanatory variable was the tertile of LOR (low/medium/high). We built separate models for each of the five subscales in ASQ and one model for ASQ-SE.

Ethical considerations

All participants provided informed written consent before inclusion in the study. The study was conducted in accordance with the Declaration of Helsinki and Good Clinical Practice guidelines. It was approved by the Ethical Committee for Research on Human Subjects at MGM Institute of Health Sciences (Ref No PVCR:2015-16:178, dated 14 May 2015, and MGIMHS/RES/02/2020-21/64, dated 20 April 2020).

## Results

Baseline characteristics

The median (IQR) range of radiation in all the households was 8.66 (IQR: 1.58, 23.11) mW/m2. It was 0.62 (IQR: 0.43, 1.58) mW/m2 in the lowest tertile, 8.66 (IQR: 5.00, 10.78) mW/m2 in the middle tertile, and 32.36 (IQR: 23.11, 45.60) mW/m2 in the highest tertile (p=0.0001). The median (IQR) distance of residence from the cell phone tower was 600 m (200 m, 800 m). In our study, 57 (54.2%) mothers were in the age group of 18 to 24 years, 37 (35.2%) in the 25 to 29 years, and 11 (10.5%) were 30 years and older. The mean (SD) birth weight of the babies was 2835.6 (469.8) gms; 19.1% (20) of infants were under the weight of 2500 gms at birth. The majority of infants were females (52.4%), and 33 (31.4%) were in the upper lower/lower socio-economic status. The mean (SD) Apgar scores were 7.50 (1.37) at one minute after birth, 8.89 (1.38) at five minutes after birth, and 9.36 (1.01) at 10 minutes after birth; there was no significant difference across the three groups of radiation levels.

Descriptive analysis

**Figure 1:**
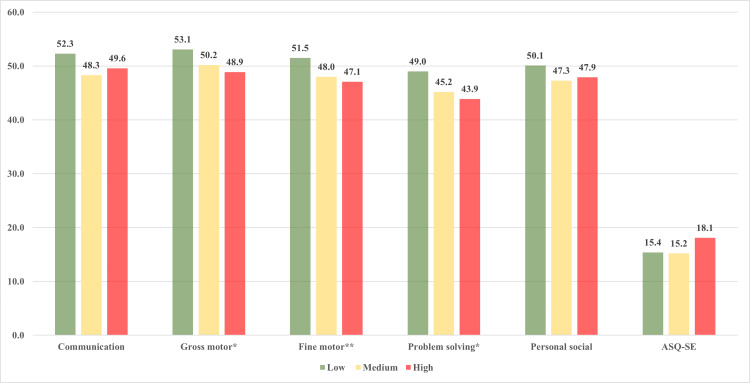
The mean scores for all domains of the Ages and Stages Questionnaire and the Ages and Stages-Social Emotional Scores according to the level of radiation * p<0.05, ** p<0.01 The X-axis represents the domain of the Ages and Stages Questionnaire, and the Y-axis represents the mean score. The three categories represent the three levels of radiation tertile: low, medium, or high.

We had 261 observations from the 105 infants. The average number of observations was 2.5 for ASQ scales and 1.5 for ASQ-SE scales (since ASQ-SE was conducted fewer times as compared with the five domains of ASQ-3). The observations for ASQ were as follows: 2 months - 96; 4 months - 54; 6 months - 38; 8 months - 25; 9 months - 16; 10 months - 15; and 12 months - 17. We have presented the findings for observations in Tables 1, 2. We found that the proportion of children classified as ‘needs referral’ (during any visit) for the ‘communication domain’ was higher in the middle (11.1%) and high (7.9%) radiation groups compared with low group (1.1%); however, the difference was not statistically significant (p=0.07). Similarly, the proportion of children classified as ‘needs referral’ for the gross motor domain was highest in the high radiation (12.4%) and middle radiation (7.4%) group compared with the low radiation group (5.5%) (p=0.40). Similar observations were also seen for the fine motor domain (Table 1). However, the difference in proportions was statistically significant for the ‘problem solving’ domain; the proportion of children classified as ‘needs referral’ was higher in the high (11.2%) and middle (12.4%) radiation group compared with the low radiation group (4.4%) (p=0.03). The difference was not significant for the ‘personal social domain’ (p=0.21) (Table 2). We found that the proportion of children classified as ‘needs referral’ for the ASQ-SE questionnaire was maximum in the high radiation group (11.2%), followed by the middle radiation group (2.2%), and none in the low radiation group (0%) (p=0.048) (Table 2). The proportions of outcomes for various demographic and clinical characteristics have been presented in Tables 1, 2. The mean scores were significantly different for the gross motor (p=0.02), fine motor (p=0.007), and problem-solving (p=0.03) domains (Figure 1). In these domains, the mean scores were significantly lower in the highest radiation tertile as compared with the lowest tertile.

**Table 1:** The outcomes for the ‘communication’, ‘gross motor’, and ‘fine motor’ domains (of the Ages and Stages Questionnaire) according to the levels of radiation in the house and other characteristics ** p<0.01, § p<0.10 The values in the parentheses represent percentages. The Total column shows the column percentages, and the other columns in each domain (normal, monitor, and refer) show the row percentages.

Parameters	Total	Communication domain			Gross motor domain			Fine motor domain		
	N (%)	Normal, n (%)	Monitor, n (%)	Refer, n (%)	Normal, n (%)	Monitor, n (%)	Refer, n (%)	Normal, n (%)	Monitor, n (%)	Refer, n (%)
	261 (100)	224 (85.8)	20 (7.7)	17 (6.5)	211 (80.8)	28 (10.7)	22 (8.4)	217 (83.1)	26 (10.0)	18 (6.9)
Radiation tertile										
Low	91 (34.9)	82 (90.1)	8 (8.8)	1 (1.1)	78 (85.7)	8 (8.8)	5 (5.5)	81 (89.0)	7 (7.7)	3 (3.3)
Middle	81 (31.0)	66 (81.5)	6 (7.4)	9 (11.1)	64 (79.0)	11 (13.6)	6 (7.4)	69 (85.2)	6 (7.4)	6 (7.4)
High	89 (34.1)	76 (85.4)	6 (6.7)	7 (7.9) ^§^	69 (77.5)	9 (10.1)	11 (12.4)	67 (75.2)	13 (14.6)	9 (10.1)
Distance from cell phone tower (meters)										
0-300	106 (40.6)	91 (85.9)	8 (7.6)	7 (6.6)	82 (77.4)	11 (10.4)	13 (12.3)	86 (81.1)	12 (11.3)	8 (7.6)
>=301	155 (59.4)	133 (85.8)	12 (7.7)	10 (6.5)	129 (83.2)	17 (10.9)	9 (5.8)	131 (84.5)	14 (9.0)	10 (6.5)
Maternal age (years)										
18-24	132 (50.6)	110 (83.3)	14 (10.6)	8 (6.1)	106 (80.3)	16 (12.1)	10 (7.6)	111 (84.1)	14 (10.6)	7 (5.3)
25-29	102 (39.1)	92 (90.2)	5 (4.9)	5 (4.9)	82 (80.4)	9 (8.8)	11 (10.8)	84 (82.4)	8 (7.8)	10 (9.8)
>=30	27 (10.3)	22 (81.5)	1 (3.7)	4 (14.8)	23 (85.2)	3 (11.1)	1 (3.7)	22 (81.5)	4 (14.8)	1 (3.7)
Birth weight (grams)										
Up to 2499	37 (14.2)	33 (83.8)	2 (5.4)	4 (10.8)	23 (62.2)	5 (13.5)	9 (24.3)	23 (62.2)	5 (13.5)	9 (24.3)
>=2500	224 (85.8)	193 (86.2)	18 (8.0)	13 (5.8)	188 (83.9)	23 (10.3)	13 (5.8)**	194 (86.6)	21 (9.4)	9 (4.0)**
Gender of baby										
Female	121 (46.4)	106 (87.6)	5 (4.1)	10 (8.3)	99 (81.8)	12 (9.9)	10 (8.3)	101 (83.5)	11 (9.1)	9 (7.4)
Male	140 (53.6)	118 (84.3)	15 (10.7) ^§^	7 (5.0)	112 (80.0)	16 (11.4)	12 (8.6)	116 (82.9)	15 (10.7)	9 (6.4)
Socio-economic status										
Upper/upper middle	72 (27.6)	60 (83.3)	6 (8.3)	6 (8.3)	61 (84.7)	9 (12.5)	2 (2.8)	65 (90.3)	3 (4.2)	4 (5.6)
Lower middle	116 (44.4)	104 (89.7)	8 (6.9)	4 (3.5)	92 (79.3)	16 (13.8)	8 (6.9)	90 (77.6)	17 (14.7)	9 (7.8)
Upper lower/lower	73 (27.9)	60 (82.2)	6 (8.2)	7 (9.6)	58 (79.5)	3 (4.1)	12 (16.4)**	62 (84.9)	6 (8.2)	5 (6.9)

**Table 2:** The outcomes for the ‘problem solving’ and ‘personal-social’ domains (of the Ages and Stages Questionnaire) and the Ages and Stages-Social Emotional Questionnaire according to the levels of radiation in the house and other characteristics * p<0.05, ** p<0.01, § p=0.053 ^a^ The total number of observations in the Ages and Stages Questionnaire-Social Emotional (ASQ-SE) was lower due to the lower frequency of follow-ups for this questionnaire. The values in parentheses represent percentages. The Total column shows the column percentages and the other columns in each domain (normal, monitor, and refer) show the row percentages.

Parameters	Total	Problem Solving			Personal Social			ASQ Social-Emotional Scores^a^			
	N (%)	Normal, n (%)	Monitor, n (%)	Refer, n (%)	Normal	Monitor, n (%)	Refer, n (%)	Total, n (%)	Normal, n (%)	Monitor, n (%)	Refer, n (%)
	261 (100)	204 (78.2)	33 (12.6)	24 (9.2)	209 (80.1)	32 (12.3)	20 (7.7)	153 (100)	137 (89.5)	9 (5.9)	7 (4.6)
Radiation tertile											
Low	91 (34.9)	81 (89.0)	6 (6.6)	4 (4.4)	80 (87.9)	6 (6.6)	5 (5.5)	55 (35.9)	52 (94.6)	3 (5.5)	0 (0.0)
Middle	81 (31.0)	58 (71.6)	13 (6.1)	10 (12.4)	61 (75.3)	12 (14.8)	8 (9.9)	46 (30.1)	41 (89.1)	4 (8.7)	1 (2.2)
High	89 (34.1)	65 (73.0)	14 (15.7)	10 (11.2) *	68 (76.4)	14 (15.7)	7 (7.9)	52 (33.9)	44 (84.6)	2 (3.9)	6 (11.5)*
Distance from cell phone tower (meters)											
0-300	106 (40.6)	83 (78.3)	13 (12.3)	10 (9.4)	82 (77.4)	16 (15.1)	8 (7.6)	65 (42.5)	55 (84.6)	5 (7.7)	5 (7.7)
>=301	155 (59.4)	121 (78.1)	20 (12.9)	14 (9.0)	127 (81.9)	16 (10.3)	12 (7.7)	88 (57.5)	82 (93.2)	4 (4.6)	2 (2.3)
Maternal age (yrs)											
18-24	132 (50.6)	102 (77.3)	20 (15.2)	10 (7.6)	100 (75.8)	21 (15.9)	11 (8.3)	78 (50.9)	72 (92.3)	1 (1.3)	5 (6.4)
25-29	102 (39.1)	81 (79.4)	11 (10.8)	10 (9.8)	86 (84.3)	9 (8.8)	7 (6.9)	58 (37.9)	48 (82.8)	8 (13.8)	2 (3.5)
>=30	27 (10.3)	21 (77.8)	2 (7.4)	4 (14.8)	23 (85.2)	2 (7.4)	2 (7.4)	17 (11.1)	17 (100.0)	0 (0.0)	0 (0.0) *
Birth weight (gms)											
Up to 2499	37 (14.2)	21 (56.8)	8 (21.6)	8 (21.6)	26 (70.3)	7 (18.9)	4 (10.8)	24 (15.7)	19 (79.2)	4 (16.7)	1 (4.2)
>=2500	224 (85.8)	183 (81.7)	25 (11.2)	16 (7.1)**	183 (81.7)	25 (11.2)	16 (7.1)	129 (84.3)	118 (91.5)	5 (3.9)	6 (4.7) ^§^
Gender of baby											
Female	121 (46.4)	97 (80.2)	14 (11.6)	10 (8.3)	97 (80.2)	17 (14.1)	7 (5.8)	74 (48.4)	67 (90.5)	4 (5.4)	3 (4.1)
Male	140 (53.6)	107 (76.4)	19 (13.6)	14 (10.0)	112 (80.0)	15 (10.7)	13 (9.3)	79 (51.6)	70 (88.6)	5 (6.3)	4 (5.1)
Socio-economic status											
Upper/Upper Middle	72 (27.6)	58 (80.6)	9 (12.5)	5 (6.9)	55 (76.4)	14 (19.4)	3 (4.2)	41 (26.8)	37 (90.2)	2 (4.9)	2 (4.9)
Lower Middle	116 (44.4)	92 (79.3)	11 (9.5)	13 (11.2)	95 (81.9)	13 (11.2)	8 (6.9)	66 (43.1)	60 (90.9)	3 (4.6)	3 (4.6)
Upper Lower/Lower	73 (27.9)	54 (73.9)	13 (17.8)	6 (8.2)	59 (80.8)	5 (6.9)	9 (12.3) ^§^	46 (30.1)	40 (86.9)	4 (8.7)	2 (4.4)

Multivariate analysis

In the random effects logistic regression models, after adjusting for distance from the cell phone tower, maternal age, birth weight, gender of the baby, and socio-economic status, we found that the odds of children classified as ‘monitor/refer’ was significantly higher in the ‘high radiation group’ compared with the ‘low radiation group’ for fine motor (aOR: 2.74, 95% CI: 1.10, 6.78; p=0.03) and problem-solving domains (aOR: 3.67, 95% CI: 1.41, 9.55; p=0.008). Similarly, we found that the odds of children classified as ‘monitor/refer’ was higher in the ‘middle radiation group’ compared with ‘low radiation group’ for problem solving (aOR: 3.12, 95% CI: 1.22, 8.00; p=0.017) and personal social (aOR: 2.67, 95% CI: 0.95, 7.50; p=0.062). We also found that low birth weight babies were significantly more likely to be classified as ‘monitor/refer’ for the fine motor (aOR: 4.19, 95% CI: 1.73, 10.14; p=0.001), and problem-solving (aOR: 2.57, 95% CI: 1.08, 6.13; p=0.033) domains. There was no significant association between distance from the cell phone tower and any of these domains. Gender, socio-economic status, and maternal age (in general) were not associated with adverse outcomes for these domains. We have presented adjusted ORs for all domains in the ASQ and ASQ-SE questionnaire in Table 3.

**Table 3:** The random effects multivariate logistic regression models for the ‘monitor/refer’ outcomes across various domains of the Ages and Stages Questionnaire and the Ages and Stages-Social Emotional Questionnaire * p<0.05, **p<0.01, § p=0.069, §§ p=0.062 aOR: adjusted odds ratio, CI: confidence intervals The values show the aORs with their 95% confidence intervals in parentheses.

Domain	Communication, aOR (95% CI)	Gross Motorm aOR (95% CI)	Fine Motor, aOR (95% CI)	Problem Solving, aOR (95% CI)	Personal Social, aOR (95% CI)	Social-Emotional^ a^, aOR (95% CI)
Radiation tertile						
Low	Reference	Reference	Reference	Reference	Reference	Reference
Middle	2.31 (0.74, 7.18)	1.45 (0.50, 4.22)	1.11 (0.41, 3.00)	3.13 (1.22, 8.00)*	2.67 (0.95, 7.50) ^§§^	1.89 (0.40, 9.00)
High	1.93 (0.61, 6.17)	1.61 (0.55, 4.70)	2.74 (1.10, 6.78)*	3.67 (1.41, 9.55)**	2.04 (0.73, 5.70)	3.41 (0.71, 16.24)
Distance from cell phone tower (meters)						
Per 100 meter increase	1.03 (0.89, 1.20)	1.01 (0.88, 1.16)	1.05 (0.94, 1.19)	1.07 (0.95, 1.19)	0.96 (0.85, 1.10)	0.98 (0.80, 1.19)
Maternal age (years)						
18-24	Reference	Reference	Reference	Reference	Reference	Reference
25-29	0.55 (0.21, 1.43)	0.99 (0.41, 2.41)	1.21 (0.56, 2.59)	0.94 (0.45, 1.94)	0.57 (0.25, 1.31)	3.19 (1.01, 10.13)*
>=30	0.77 (0.17, 3.48)	0.78 (0.17, 3.55)	2.29 (0.67, 7.80)	0.91 (0.27, 3.12)	0.42 (0.09, 1.90)	--
Birth weight (grams)						
>=2500	Reference	Reference	Reference	Reference	Reference	Reference
Up to 2499	0.94 (0.28, 3.15)	2.71 (0.93, 7.87) ^§^	4.19 (1.73, 10.14)**	2.57 (1.08, 6.13)*	1.57 (0.57, 4.32)	2.48 (0.63, 9.74)
Gender of baby						
Male	Reference	Reference	Reference	Reference	Reference	Reference
Female	1.42 (0.56, 3.58)	1.24 (0.52, 2.97)	0.88 (0.41, 1.90)	1.33 (0.65, 2.73)	0.91 (0.41, 2.03)	1.73 (0.53, 5.69)
Socio-economic status						
Upper Lower/Lower	Reference	Reference	Reference	Reference	Reference	Reference
Lower Middle	0.40 (0.13,1.22)	1.07 (0.38, 3.05)	1.78 (0.71, 4.49)	0.57 (0.24, 1.36)	0.96 (0.36, 2.59)	0.48 (0.11, 2.07)
Upper/Upper Middle	0.59 (0.18, 1.93)	0.74 (0.22, 2.49)	0.67 (0.21, 2.12)	0.54 (0.21, 1.40)	1.28 (0.43, 3.77)	0.80 (0.17, 3.90)

## Discussion

In this cohort study, we found that mean ASQ scores were, in general, lower in the highest radiation tertile for all five domains; and specifically, significantly lower for the gross motor, fine motor, and problem-solving domains. Poor development outcomes (such as monitor/refer) for the fine motor, problem-solving, and personal-social domains were associated with higher levels of radiation in the house. In addition, low birth weight was also significantly associated with poor development outcomes for fine motor and problem-solving. Finally, even though mean scores for the social-emotional domain were high in the higher radiation groups, the difference was not statistically significant.

EMF radiations are present in the environment, and with an increase in the use of wireless services, the exposure to these radiations is going to increase and will be a major topic of research and discussion. These two types of EMF radiations - the extremely low frequency EMF radiations (ELF-EMF) and radiofrequency EMF radiations (RF-EMF) - have numerous health effects [32]. Some of the main symptoms of electromagnetic hypersensitivity are sleep disorders, headache, back pain, sweating, body rash, depression, and loss of energy. However, as indicated earlier, numerous authors have also studied the role of these EMF radiations on developmental delays in children and adolescents. In our cohort, we found that a specific developmental domain - the cognitive domain - was, in general, more affected in neonates and infants. A study by Choi and colleagues did not find any significant association between prenatal exposure to radiofrequency radiation and child neurodevelopment [33]. These researchers used the mental developmental index and psychomotor development index of the Bayley Scales of Infant Development-Revised tool [33]. Other studies have primarily assessed the role of mobile phone use and developmental delays. In the Danish National Birth Cohort study, the authors did not find any significant association between prenatal cell phone use and motor and cognitive/language delays [19]. In another cohort study from Spain, the authors found that the developmental scores did not follow a particular pattern in prenatal cell phone users [22]. On one hand, the mental development scores were higher in cell phone users, whereas on the other hand, the scores were lower for the psychomotor development scale. However, another meta-analysis reported that fetal development disorders and childhood development disorders were higher in parents who were exposed to EMFs compared with those who had not [34]. Cabré-Riera and colleagues studied the association between whole brain RF-EMF dose and cognitive function; they found that a higher exposure to RF-EMF may be associated with lower non-verbal intelligence but not other components of cognitive function [35]. Other studies have assessed cognitive function in adolescents. For instance, Bhatt and colleagues found little evidence that the use of mobile phones or cordless phones affected cognitive function in primary school children [36]. Guxens et al. studied both the effects of RF-EMF exposure and the use of mobile phones or cordless phones in children. They reported that five-year-old children who were exposed to higher radiation from mobile base stations were more likely to have parent-reported emotional problems. However, mobile phones/cordless phones were not associated with behavioural problems in children [37]. Bodewein and colleagues, after having reviewed epidemiological and experimental studies, reported low to inadequate evidence of the effects of RF-EMF or mobile communication devices on behavior and cognition in children [38].

Another important factor associated with developmental issues was weight at birth. We did find that low birth weight infants had a higher likelihood of poorer outcomes for fine motor and problem-solving domains. Previous studies have also highlighted the relationship between low birth weight and developmental delays. A study by Hilaire et al. reported that normal birth weight babies had higher scores for gross motor, cognitive, and expressive communication skills compared with low birth weight babies [39]. Another study from Rwanda found that low birth weight and/or preterm babies were significantly more likely to have developmental delays [40]. Some authors also reported that very low birth weight babies have higher neurodevelopmental impairments [41]. In the present study, even after adjusting for radiation levels, low birth weight was associated with motor developmental delays.

There are potential limitations in the present analysis. In this study, the focus was on the association between the levels of electromagnetic field radiation and neurodevelopmental outcomes in neonates and infants. Other studies have included prenatal cell phone use and exposure as the exposure variable; we did not include these in the present analysis. We also did not assess the parent-child interaction as another potential confounder. The EMF radiation in the house may not only be due to that from cell phone towers but also due to cordless phones, WiFi devices, and Bluetooth gadgets. Since we measured the radiation in the house, this would have included the EMF from all these. It is quite likely that a home far away from the tower may still have higher EMF radiation due to these gadgets. However, the measurement was only at baseline, and this may have led to some misclassification. As described earlier, some authors have not found an association between cell phone use, while others have found inconsistencies in the relationship. Most other authors have used other scales, such as the Bayley Scales of Infant Development or scales that were developed by the researcher. We have used the Ages and Stages Questionnaire, both for the developmental domain and the social-emotional domain. ASQ is useful for screening and not a diagnostic instrument. We also do not claim causality in these findings. These are interim results of the cohort, and we are still following the cohort. We intend to publish future results on the neurodevelopmental outcomes. These outcomes will include the Bayley Scales of Infant Development and the Stanford-Binet test for intelligence. We did not use any imputation methods for missing observations and just used the random effects models with available observations. Though we had enough power to detect the difference in scores, some of the events were few (particularly social-emotional and communication domains). Thus, we may be underpowered at this point of analysis for these outcomes. Nonetheless, actual measurement of RF-EMF radiation in the house (which represents the actual exposure levels), a cohort design, and the use of random effects logistic regression models are the potential strengths of the study. These models are useful for longitudinal data, and one additional advantage of random effects models is their use in longitudinal data when observations are present at different time points [42,43].

## Conclusions

These preliminary findings are an important contribution to the literature on the association between RF-EMF radiation and neurodevelopment in neonates and infants. The results have to be interpreted taking into account the limitations that have been mentioned. We used random effects models, which account for both within-subject and between-subject correlation, and are useful for time-varying variables. These models are useful for longitudinal data where the outcomes may vary with each observation. Even after adjusting for low birth weight, we found that higher levels of radiation were associated with poorer outcomes for cognitive domains of development such as the problem-solving and personal-social areas. Low birth weight was associated with poorer outcomes for the motor development domains (gross motor and fine motor). It is quite likely that obvious gross motor delays may be identified by parents and caregivers; however, cognitive development domains require specialist monitoring. Thus, there may be a need to consider monitoring of neurodevelopmental outcomes in children in whom RF-EMF radiations are expected to be higher (such as very close to cell phone towers and too many gadgets in the house).
